# Action Mechanisms of Exosomes Derived from GD3/GD2-Positive Glioma Cells in the Regulation of Phenotypes and Intracellular Signaling: Roles of Integrins

**DOI:** 10.3390/ijms252312752

**Published:** 2024-11-27

**Authors:** Mohammad Abul Hasnat, Yuhsuke Ohmi, Farhana Yesmin, Kei Kaneko, Mariko Kambe, Yoko Kitaura, Takako Ito, Yuka Imao, Keiko Kano, Emi Mishiro-Sato, Hiroka Koyanagi, Yoshiyuki Kawamoto, Robiul Hasan Bhuiyan, Yuki Ohkawa, Orie Tajima, Koichi Furukawa, Keiko Furukawa

**Affiliations:** 1 Department of Biomedical Sciences, Chubu University, Matsumoto 1200, Kasugai 487-8501, Aichi, Japan; lalon.hasnat@gmail.com (M.A.H.); farhana7779@gmail.com (F.Y.); ikekoneka@isc.chubu.ac.jp (K.K.); kambem@isc.chubu.ac.jp (M.K.); imao@isc.chubu.ac.jp (Y.I.); rb23004-0529@sti.chubu.ac.jp (H.K.); ykawa@isc.chubu.ac.jp (Y.K.); oriet@isc.chubu.ac.jp (O.T.); 2 Department of Biochemistry and Molecular Biology, Shahjalal University of Science and Technology, Sylhet 3114, Bangladesh; 3 Department of Clinical Engineering, Chubu University College of Life and Health Sciences, Kasugai 487-8501, Aichi, Japan; ooumi82@isc.chubu.ac.jp (Y.O.); ykitaura@isc.chubu.ac.jp (Y.K.); taca1021@isc.chubu.ac.jp (T.I.); 4 WPI-ITbM (Institute of Transformative Bio-Molecules), Nagoya University, Nagoya 464-8601, Aichi, Japan; kano.keiko.f4@f.mail.nagoya-u.ac.jp (K.K.); mishiro.emi.m5@f.mail.nagoya-u.ac.jp (E.M.-S.); 5 Department of Biochemistry and Molecular Biology, Faculty of Biology, Chittagong University, Chittagong 4331, Bangladesh; biochemistrobi79@gmail.com; 6 Lab of Cancer Glycobiology, Osaka Cancer Center, Osaka 541-8567, Osaka, Japan; yuki.ohkawa@oici.jp

**Keywords:** ganglioside, exosome, extracellular vesicle, glioma, integrin

## Abstract

Extracellular vesicles (EVs) play important roles in intercellular communication in various biological events. In particular, EVs released from cancer cells have attracted special attention. Although it has been reported that cancer-associated glycosphingolipids play important roles in the enhancement of malignant properties of cancer cells, the presence, behavior, and roles of glycosphingolipids in EVs have not been elucidated. Recently, we reported crucial roles of EVs expressing gangliosides, GD2, and/or GD3 in the enhancement of cancer properties in malignant melanomas and gliomas. However, how EVs containing cancer-associated glycosphingolipids play their roles has not been reported to date. Here, we studied spatio-temporal mechanisms for GD3/GD2-containing EVs released from gliomas in the actions toward target cells. Proteome analyses of EVs with/without GD3/GD2 revealed an equally high concentration of integrin isoforms in both GD3/GD2+ and GD3/GD2- EVs. PKH26-labeled EVs attached, invaded, and distributed to/in the target cells within 1 h. GD3/GD2 formed molecular complexes with integrins on EVs as elucidated by immunoprecipitation/immunoblotting and immunocytostaining. The addition of antibodies reactive with GD3, GD2, or integrins resulted in the suppression of the enhancing effects of EVs in the cell adhesion assay. The addition of GD3/GD2 + EVs to GD3/GD2- cells clearly increased the phosphorylation levels of the PDGF receptor, FAK, and Erk1/2 in immunoblotting, suggesting GD3/GD2+ EVs activate the signaling pathway in the target cells within 15 min after addition. Anti-ganglioside antibodies clearly blocked signaling with EVs. In conclusion, EVs released from GD3/GD2-expressing glioma cells enhance cancer phenotypes and malignant signals via the cluster formation of integrins and GD3/GD2 on EVs, leading to the regulation of the cancer microenvironment.

## 1. Introduction

Gangliosides, sialic acid-containing glycosphingolipids, have been reported to be dominantly expressed in the nervous systems of vertebrates [1], while almost all cells and tissues in our bodies also express some kinds of gangliosides [2]. Among gangliosides, mature-type gangliosides with an extended core structure such as GM1, GD1a, GD1b, and GT1b are expressed in developed brain tissues [3]. On the other hand, relatively simple structures such as GD3 and GM3 have been reported to be expressed at the early stage of neurogenesis in infantile nervous systems [3].

Glioma is one of the brain tumors occurring from glial cells or their precursor cells [4], exhibiting highly malignant phenotypes and grim prognosis for patients. In these gliomas, it has been reported that simple structured gangliosides such as GD3 and GD2 are considered to be cancer-associated antigens [5,6,7,8,9,10] as in other neuroectoderm-derived tumors like malignant melanomas and neuroblastomas [11,12,13,14]. Not only neuroectoderm-derived tumors but many other cancers also express GD3 and/or GD2, e.g., T-cell leukemias [15,16], small cell lung cancers [17,18], osteosarcomas [19] retinoblastomas [20], and breast cancers [21,22]. Many of these studies reported that the expression of disialyl gangliosides increases the malignant degree of tumors [23,24,25,26] and brings about difficulties in the treatment of patients [27].

Recently, extracellular vesicles (EVs) named exosomes have been paid strong attention to, since the importance of their roles in intercellular communication has been reported [28,29]. In particular, unique functions of exosomes released from various cancer cells have been reported, e.g., the regulation of tumor microenvironments [30] and niche formation for cancer metastasis [31,32]. Exosomes are released from various normal and malignant cells, providing valuable information for disease diagnosis [33,34]. The roles of various molecules contained in individual exosomes, such as DNA, mRNA, microRNA, lipids, cytoplasmic proteins, and membrane proteins, have been rigorously analyzed, and their therapeutic applications are being discussed [35]. However, the presence and significance of cancer-associated glycosphingolipids in exosomes has scarcely been analyzed or reported to date.

Recently, we have reported the expression and function of the ganglioside GD2 in malignant melanoma cells [36], since we have studied functions of these cancer-associated gangliosides in various malignant tumor cells such as melanomas [24,26], SCLCs [18], osteosarcomas [19], and leukemias [37]. We also reported roles of GD3/GD2 in the malignant phenotypes of gliomas using transfectant cells of *ST8SIA1* cDNA [38]. Then, we reported the roles of exosomes released from GD2-expressing melanoma cells in the enhancement of malignant properties and signaling of GD2-negative target cells [39]. As for exosomes released from glioma cells, we have briefly reported the crucial roles of GD3/GD2-expressing cell-derived exosomes in the enhancement of malignant phenotypes [40]. However, it is not understood at all how exosomes access and modulate the phenotypes and intracellular signaling of target cells. In this study, we analyzed the comprehensive composition of exosomes from GD3/GD2-expressing glioma cells and spatio-temporal mechanisms for the actions of exosomes toward and in target cells. The observed results should give us important insights into the understanding of exosome actions and also the application of exosomes for cancer therapeutics.

## 2. Results

The effects of GD3/GD2-expressing glioma cell-derived exosomes on phenotypes were briefly reported in a previous paper [40]. Therefore, the mechanisms of exosome actions are analysed and reported here. Based on these past experiments, appropriate doses of the exosomes to be added were decided.

### 2.1. Proteome Analysis of EVs Released from GD3/GD2(+) and GD3/GD2(-) Glioma Cells

To compare the contents of exosomes released from GD3/GD2(+) cells and GD3/GD2(-) cells, proteome analysis was performed at the Nagoya University Institute of Transformative Bio-Molecules (ITbM) laboratory.

**Figure 1:**
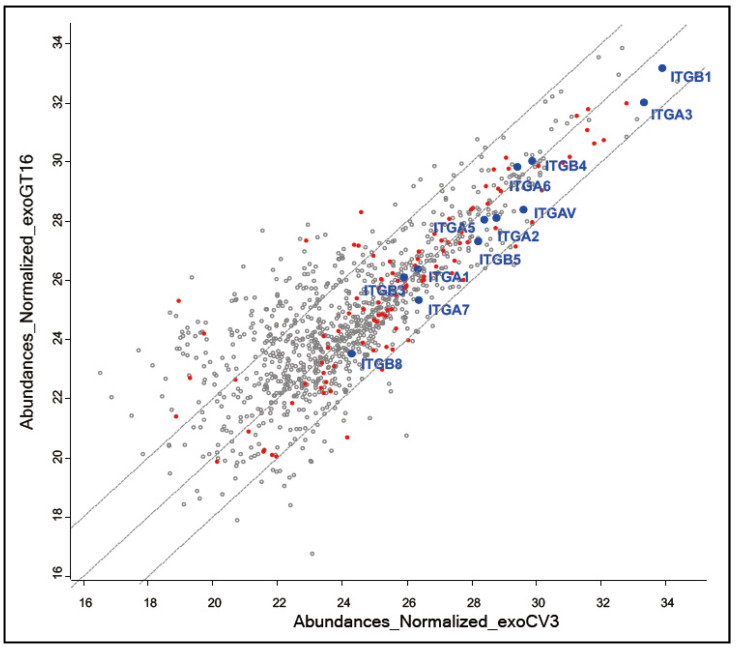
EVs from GT16 and CV3 were analyzed by MS. The abundance of proteins was compared after normalization based on the total peptide amount according to the human database of Discoverer 2.4. Perseus analysis was performed after focusing on Master Protein, removing contamination, and taking higher than PSMs 4. In the scatter plot, the X-axis represents the log2-transformed normalized abundance of CV3-derived EVs, while the Y-axis represents the log2-transformed normalized abundance of GT16-derived EVs. The plot includes fold change lines at ±2 log2 unit. These lines indicate a 4-fold increase (+2 log2 unit) or a 0.25-fold decrease (−2 log2 unit) in the expression level of GT16-derived EVs relative to CV3-derived EVs. Integrin binding proteins are marked by red circles based on the GO term. Integrin isoforms are marked by blue dots. Other identified proteins are indicated by gray circles.

In proteome analysis, 1053 proteins were identified in EVs from GT16 and CV3, as shown in Figure 1. While 643 EV-related proteins were detected, it was characteristic that integrin β1 was the most frequently found protein, and other integrin isoforms were also enriched (blue dots). In GO terms, 54 integrin binding proteins were found (red circle), indicating that integrin-related proteins were highly concentrated in the prepared EVs.

Between EVs from GD3/GD2+ GT16 and GD3/GD2- CV3, integrin β1 and α3 showed a mild increase in exoCV3 in abundance, but no marked differences in the ratio between two samples could be found. Other isoforms of integrins could be also found with no clear differences in the ratio, i.e., all integrin isoforms could be found within a 4-fold increase/decrease [16].

### 2.2. Time Course of Expression of Gangliosides and Ganglioside Synthase Genes in the Recipient Cells After Treatment with EVs from GD3/GD2-Expressing Cells

#### 2.2.1. Expression of Gangliosides GD3 and GD2 on the Surface of GD3/GD2-Negative Cells After Treatment with EVs Derived from GD3/GD2-Expressing Cells

**Figure 2:**
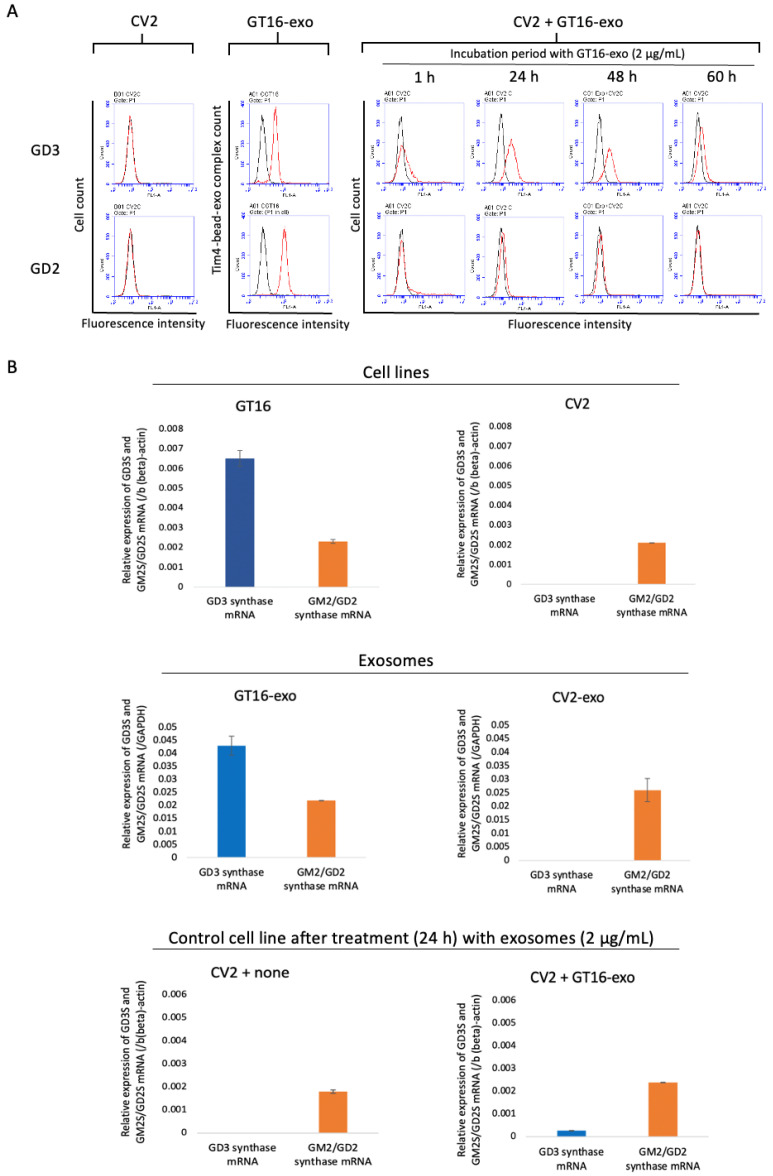
Time course of expression of gangliosides and ganglioside synthase genes on/in the CV2 (control) cells after treatment with EVs from GD3/GD2-positive cells. The expression of gangliosides on EVs released from the GD3/GD2(+) clone GT16 was analyzed using Tim4-beads flow cytometry (**A, left**). The cell surface expression of GD3 and GD2 on the GD3/GD2(-) clone CV2 was analyzed before and after treatment with GD3/GD2(+) cell-derived EVs with different time courses by flow cytometry (**A, right**). Red lines are results with individual mAbs, and black lines are of negative controls. Using RT-qPCR, the expression of GD3 synthase mRNA and GM2/GD2 synthase mRNA was examined in both cell lines (**B, top**) and EVs (**B, middle**) released from them. These mRNAs in the GD3/GD2(-) clones at 24 h after treatment with GD3/GD2(+) clone-derived EVs (2 μg/mL) were also analyzed (**B, bottom**).

GD3/GD2(+) cell lines (e.g., GT16) were established by transfecting a human glioma cell line, U-251MG (GD3/GD2-non-expressing), with GD3 synthase cDNA. The GD3/GD2(-) cell lines (e.g., CV2) were generated as vector controls [38]. The time course of expression of gangliosides GD3 and GD2 on control CV2 after treatment with GT16-derived exosomes is shown in Figure 2A. The definite expression of GD3 was observed on CV2’s cell surface after 24 h to 48 h of the EV treatment, but only minimal expression of GD2 was observed at 1~48 h after the EV treatment by flow cytometry.

#### 2.2.2. Expression of GD3 Synthase and GM2/GD2 Synthase mRNAs as Analyzed by RT-qPCR

GD3/GD2(+) and GD3/GD2(-) cell lines, with exosomes released from both of them, and GD3/GD2(-) cells after treatment (24 h) with GD3/GD2(+) cell-derived EVs were analyzed to examine the expression levels of GD3 synthase and GM2/GD2 synthase mRNAs. In the case of GD3/GD2(+) cells and the EVs derived from them, both GD3 synthase mRNA and GM2/GD2 synthase mRNA were observed, but in GD3/GD2(-) cells and in EVs derived from them, only GM2/GD2 synthase mRNA was expressed (Figure 2B). Surprisingly, the GD3/GD2(-) cells expressed GD3 synthase mRNA besides GM2/GD2 synthase mRNA after 24 h treatment with GD3/GD2(+) cell-derived EVs (Figure 2B).

### 2.3. Attachment and Invasion of PKH26-Labeled GD3/GD2(+) Cell-Derived EVs to/into GD3/GD2(-) Cells

**Figure 3:**
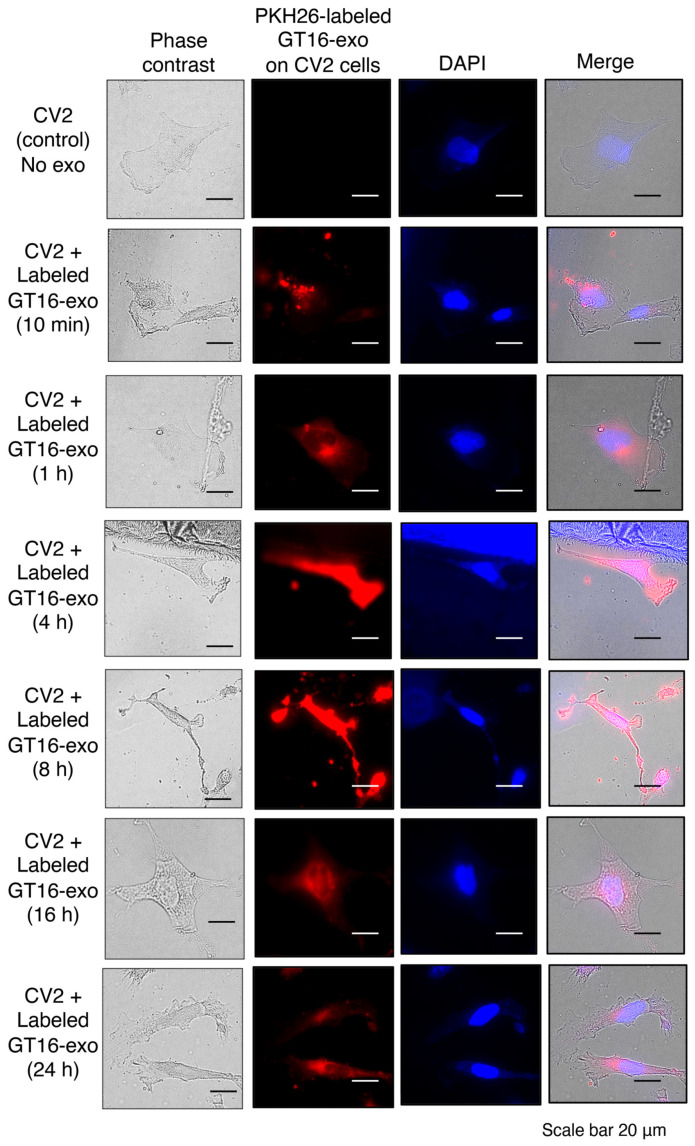
Attachment and fates of PKH26-labeled GD3/GD2(+) EVs on/in recipient GD3/GD2(-) cells. About 20 μg of GD3/GD2(+) cell-derived EVs was stained with PKH26 (0.003 mM) for 5 min, and then unbound PKH26 was washed out by repeated ultracentrifugation for 1 h at 110,000× *g* and 4 °C. The labeled EVs (2 μg/mL) were used to treat the cultured GD3/GD2(-) cells (2 × 10^4^) on collagen-I-pre-coated 6 cm dishes for 0, 10 min, 1 h, 4 h, 8 h, 16 h, and 24 h at 37 °C. After washing the unbound labeled EVs, the cells were stained with 2 μg/mL of DAPI (Fujifilm, Japan) in PBS for 20 min for nuclear staining (blue color). Subsequently, the attachment and invasion of PKH26-labeled EVs (red color) to/into GD3/GD2(-) cells were analyzed by an all-in-one fluorescence microscope, the BZ-810, KEYENCE (Tokyo, Japan) after fixation with 4% paraformaldehyde. A representative pattern from more than 10 images at each time point was shown.

The PKH26-labelled GD3/GD2(+) cell-derived EVs were used to treat the cultured GD3/GD2(-) cells. The attachment and invasion of labelled EVs to/into GD3/GD2(-) cells were analyzed by confocal microscopy after fixation. With increasing time, more attachment and invasion of labeled EVs to/into GD3/GD2(-) cells were observed (Figure 3). Ten minutes after addition to the plate, labeled EVs appeared to attach around the target cells and partially invade the cells. After 1 h, they could be found mainly in the cytoplasm. Then, 4 and 8 h after addition, EVs were distributed all over the cells in both the cytoplasm and nucleus. However, the majority of them were eventually located in the cytoplasm, mainly around the nucleus, 16 and 24 h after addition to the target cells.

### 2.4. Expression and Association of Integrins and Gangliosides on the Surface of Cells and EVs

#### 2.4.1. Expression of Integrins and Gangliosides on Cells and EVs

**Figure 4:**
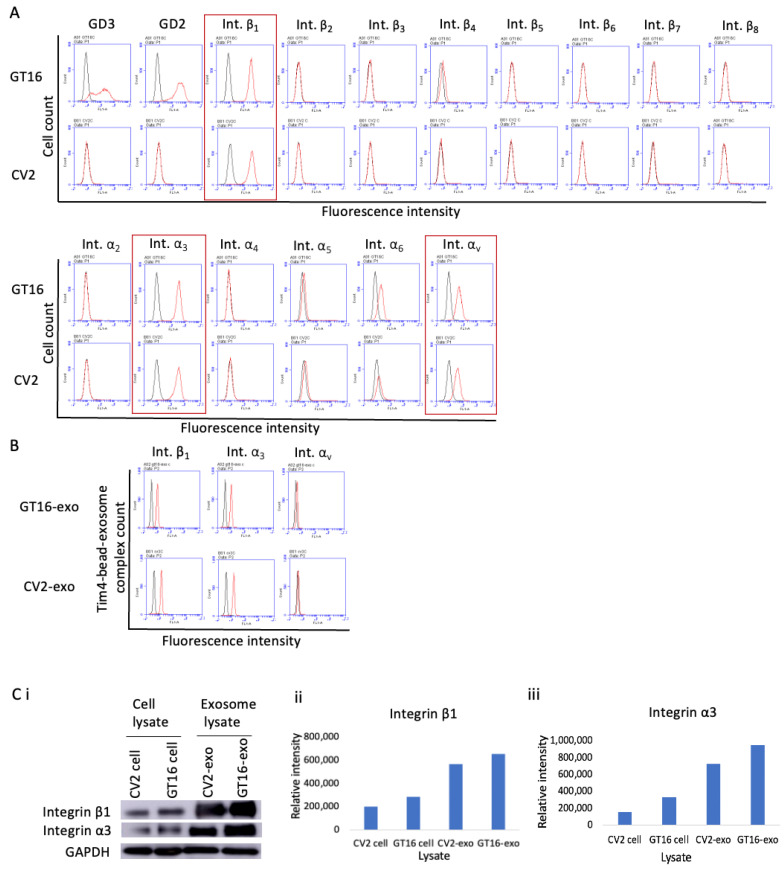
The expression of integrins and gangliosides on/in cells and EVs. Cell surface expression of gangliosides, GD3 and GD2, and that of integrins, β_1,_ β_2,_ β_3,_ β_4,_ β_5,_ β_6,_ β_7,_ β_8,_ α_2,_ α_3,_ α_4,_ α_5,_ α_6,_ and α_v_, were analyzed by flow cytometry. Anti-GD3 mAb (R24), anti-GD2 mAb (220-51), anti-integrin β_1_~β_8_ Abs, anti-integrin α_2_~α_6_ Abs, or anti-integrin α_v_ Ab were used as primary antibodies, and FITC-labeled secondary antibodies were used (**A**). The expression levels of integrin β_1,_ α_3,_ and α_v_ were analyzed in both GD3/GD2(+) and (-) cell-derived EVs by Tim4-bead flow cytometry (**B**). Red lines are results with individual mAbs, and black lines are of negative controls. The expression of integrin β_1_ and α_3_ was observed in GT16 and CV2 cells and also in EVs derived from them by IB using lysates (1 μg) of both cells and EVs. Anti-integrin β_1_ Ab and anti-integrin α_3_ Ab were used as primary antibodies, and HRP-labelled secondary Ab was used (**Ci**). The intensity of the obtained bands in Figure 4Ci was measured and plotted (**Cii,Ciii**). Representative results from repeated experiments (at least 3 times) are presented.

The expression of integrins (β_1~_β_8,_ α_2~_α_6,_ and α_v_) and gangliosides (GD3 and GD2) on GT16 and CV2 cells was observed by flow cytometry, showing the definite and equivalent expression of integrin β_1,_ α_3,_ and α_v_ in both cell lines (Figure 4A). An almost similar expression pattern of integrin β_1_ and α_3_ was observed on EVs derived from both GT16 and CV2 cells (Figure 4B). In IB, slightly higher expression of integrin β_1_ and α_3_ was detected in GT16 cells and EVs derived from them than in CV2 cells and EVs derived from them, respectively (Figure 4C). Generally, the band intensities of integrins were higher in exosome lysates than in cell lysates for both cells.

#### 2.4.2. Colocalization of Gangliosides and Integrins on Cell Surface as Shown by Double Immunostaining

**Figure 5:**
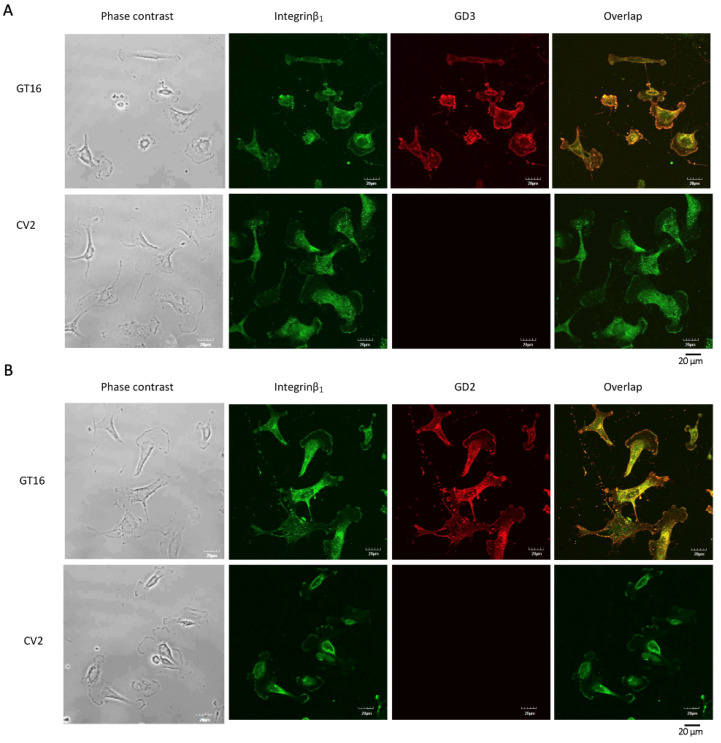
Colocalization of GD3 and/or GD2 with integrin β_1_ on the cell surface. GD3/GD2(+) GT16 and GD3/GD2(-) CV2 cells (2 × 10^4^) were seeded on collagen-I-pre-coated glass-bottom dishes and fixed on the bottom of the dish with 4% paraformaldehyde at 70 to 80% confluency. The colocalization of GD3 and integrin β_1_ was analyzed by staining with two kinds of primary antibodies, i.e., mouse anti-GD3 mAb (1:100) and rabbit anti-integrin β_1_ Ab (1:100), and then by the individual secondary antibodies, Alexa 568-conjugated goat anti-mouse IgG Ab (red) (1:100) and Alexa 488-conjugated goat anti-rabbit IgG (H+L) Ab (1:100) (green), respectively, under a confocal microscope (FLUOVIEW FV10i, Olympus, Tokyo, Japan). (**A**). In the case of the colocalization of GD2 and integrin β_1_ on the cell surface, mouse anti-GD2 mAb (1:100) was used with rabbit anti-integrin β_1_ Ab (1:100) as primary Abs, and the secondary Abs were as mentioned above (**B**).

The colocalization of integrin β_1_ with GD3 and/or with GD2 on the surface of GT16 and CV2 cells was examined by double immunostaining (Figure 5A,B). Mouse anti-GD3 mAb, mouse anti-GD2 mAb, and rabbit anti-integrin β_1_ Ab were used as primary antibodies. Alexa 568-conjugated goat anti-mouse IgG Ab (red) and Alexa 488-conjugated goat anti-rabbit IgG (H+L) Ab (green) were used as secondary Abs. The results suggested that both gangliosides, GD3 (Figure 5A, upper red) and GD2 (Figure 5B, upper red), were colocalized with integrin β_1_ on the GT16 cell surface mainly at the leading edge (Figure 5A,B, upper right, yellow).

#### 2.4.3. Close Localization of Gangliosides and Integrins on the Surface of EVs by Double Immunostaining

**Figure 6:**
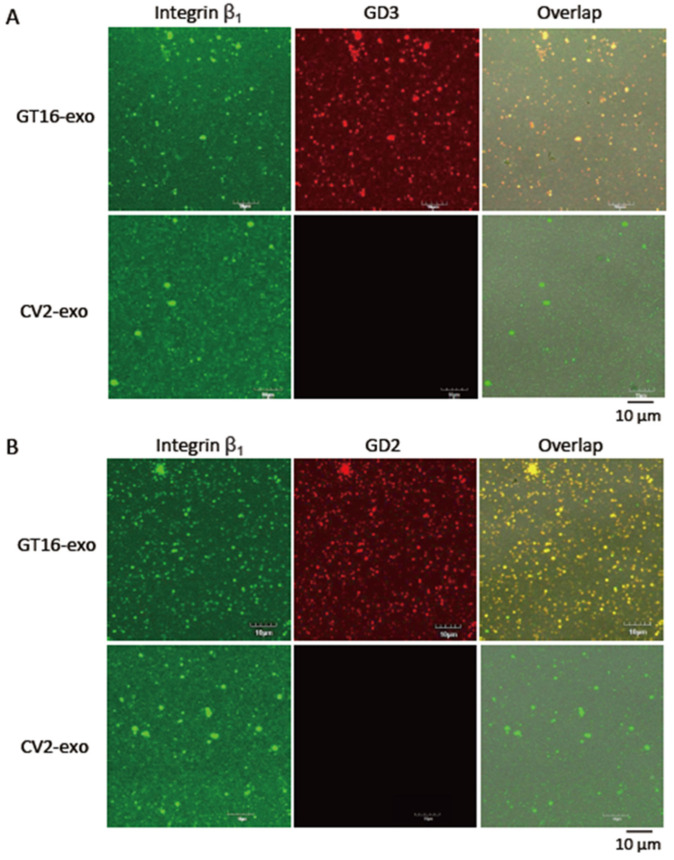
The close localization of GD3 and/or GD2 with integrin β_1_ on EVs as analyzed by double immunostaining. Fresh exosomes (3 μg/200 μL) were added on Tim4-Fc-coated glass-bottom dishes for 30 min at RT. After washing, the bound exosomes were fixed on the bottom of the dish with 4% paraformaldehyde. Fixed exosomes were incubated with primary antibodies (mouse anti-GD3 mAb (1:100) and rabbit anti-integrin β_1_ Ab (1:100)) and secondary Abs (Alexa 568-conjugated goat anti-mouse IgG Abs (red) (1:100) and/or Alexa 488-conjugated goat anti-rabbit IgG (H+L) Abs (green) (1:100)) and finally analyzed with a confocal microscope, where the yellow color of the overlapping image indicates the close localization of GD3 and integrin β_1_ on GT16-derived EVs (**A upper**) but not on CV2-derived EVs (**A lower**). Colocalization between GD2 and integrin β_1_ was examined on EVs using primary antibodies, mouse anti-GD2 mAbs (1:100) and rabbit anti-integrin β_1_ Abs (1:100), and the proper secondary Abs as mentioned above. The yellow color of the confocal imaging means close localization between GD2 and integrin β_1_ on GT16 cell-derived EVs (**B upper**), but not on CV2-derived EVs (**B lower**).

On the surface of EVs, the close localization of GD3 or GD2 with integrin β_1_ was analyzed by confocal microscopy after double immunostaining (Figure 6A,B). Like double staining the cells (Figure 5), GD3/GD2(+) and (-) cell-derived EVs were also stained with primary and secondary Abs. The expression of integrin β_1_ on EVs was marked by a green color (Figure 6, left), while the GD3 (Figure 6A, upper middle) and GD2 (Figure 6B, upper middle) were marked by a red color. After overlapping the images, a yellow color was formed (Figure 6A,B, upper right), suggesting the close association of GD3 and/or GD2 with integrin β_1_. Due to the absence of GD3 and/or GD2 on GD3/GD2(-) cell-derived EVs, a red color was not observed on them (Figure 6A,B, lower middle) and consequently, no yellow color was found (Figure 6A,B, lower right).

#### 2.4.4. Molecular Clustering of Gangliosides and Integrin β_1_ on Cells and EVs as Analyzed by IP Followed by IB

**Figure 7:**
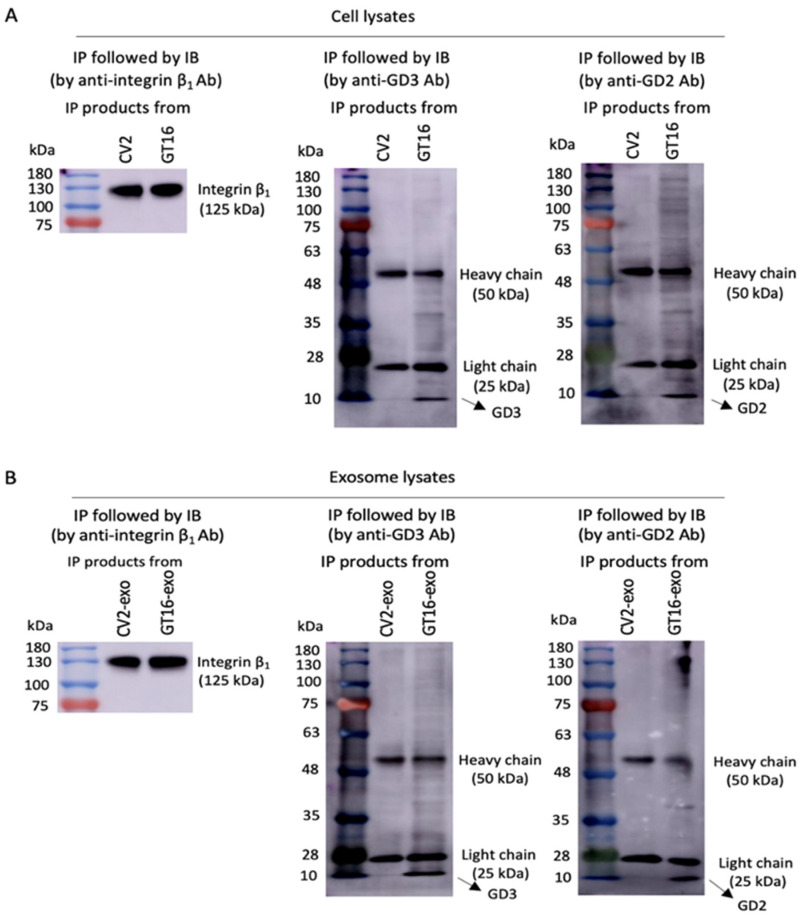
Molecular clustering of gangliosides and integrin β_1_. (**A**) The association of GD3 and integrin β_1_ and that of GD2 and integrin β_1_ on cells was analyzed by IP with anti-integrin β_1_ Ab and subsequent IB with anti-GD3 mAb or anti-GD2 mAb as well as anti-integrin β_1_ Ab. (**B**) The molecular clustering between GD3 and integrin β_1_ and between GD2 and integrin β_1_ in EVs derived from them was examined by IP followed by IB as mentioned above.

The molecular clustering of integrin β_1_ with GD3 and/or GD2 in GD3/GD2(+) and GD3/GD2(-) cells and in EVs derived from both of them was analyzed by IP and subsequent IB. Cell lysates from GD3/GD2(+) and/or GD3/GD2(-) cells as well as lysates from EVs derived from GD3/GD2(+) and/or GD3/GD2(-) cells were used for IP with the anti-integrin β_1_ antibody. Then, the products of IP were immunoblotted with anti-GD3 mAb or anti-GD2 mAb as well as anti-integrin β_1_ Ab. In the case of cell lysates, GD3 and GD2 were detected by IB from the IP complex obtained from GD3/GD2(+) cell (GT16) lysates (Figure 7A). Then, both the gangliosides GD3 and GD2 were also detected from IP products obtained from GT16 cell-derived EV lysates (Figure 7B) by IB. These results indicated that GD3 and/or GD2 form molecular clusters with integrin β_1_ on GT16 cell membrane and also on EVs derived from them.

### 2.5. Effects of Antibodies to Disialyl-Gangliosides or to Integrins on the Actions of EVs

#### 2.5.1. Effects of Anti-Integrin mAbs on EV Functions

**Figure 8:**
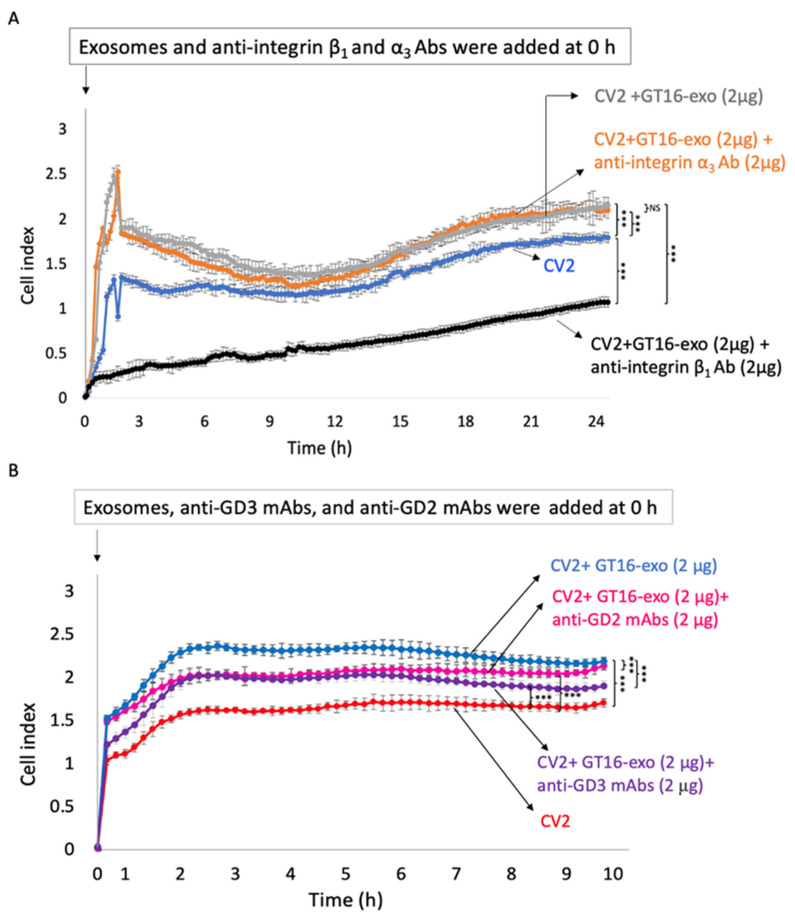
Effects of anti-integrin Abs and anti-GD3/GD2 mAbs on EV function. (**A**) The adhesion activity of GD3/GD2(-) cells to collagen-I examined by RT-CES. Cells (1 × 10^4^) were cultured in collagen-I-precoated wells containing 100 μL of culture medium and incubated. The data after 24 h (**A**) and 9 h (**B**) of cell incubation are shown. GT16 cell-derived EVs enhanced the adhesion activity of CV2 cells (**A**,**B**), but anti-integrin β_1_ Ab remarkably suppressed the adhesion activity of CV2 cells (**A**). Anti-GD3 mAb and/or anti-GD2 mAb also significantly suppressed the action of EVs to the adhesion of CV2 cells when either one of these anti-ganglioside mAbs was added to CV2 cells together with GD3/GD2(+) cell-derived EVs (**B**). The data until 24 h of incubation (**A**) and until 9 h of incubation (**B**) were analyzed with an unpaired Student’s two-tailed *t* test. Not significant (NS) *p* > 0.05, and *** *p* < 0.001. The mean values ± SD (n = 3) were plotted for each time point.

The effects of anti-integrin β_1_ Ab_,_ and anti-integrin α_3_ Ab on the adhesion of GD3/GD2(-) and CV2 cells to collagen-I in the presence of GT16 cell-derived EVs were investigated using a real-time cell electronic sensing system (RT-CES). Although GD3/GD2(+) cell-derived EVs increased the adhesion levels of CV2 cells, anti-integrin β_1_ Ab strongly suppressed the adhesion of CV2 cells, probably by binding with integrin β1, present on both CV2 cells and on EVs. On the other hand, no clear effect of anti-integrin α_3_ on cell adhesion was observed when compared with those of anti-integrin β_1_ Ab (Figure 8A).

#### 2.5.2. Effects of Anti-Ganglioside mAbs on EV Functions

The adhesion activity of CV2 cells to collagen-I as analyzed by RT-CES was increased after treatment with GT16 cell-derived EVs. The effects of anti-GD3 Ab or anti-GD2 Ab on the action of GT16 cell-derived EVs were examined. The increased adhesion activity of CV2 cells was observed after the EV treatment, but less enhanced adhesion was observed when cells were treated with either anti-GD3 mAb or anti-GD2 mAb together with GT16 cell-derived EVs (Figure 8B). These suppressive effects of anti-ganglioside mAbs seemed to be due to the blocking of gangliosides on EVs.

### 2.6. Effects of EVs on the Cell Adhesion Signal and Roles of Gangliosides

#### 2.6.1. Effects of GD3/GD2(+) EVs on GD3/GD2(-) Cell Adhesion Signaling

**Figure 9:**
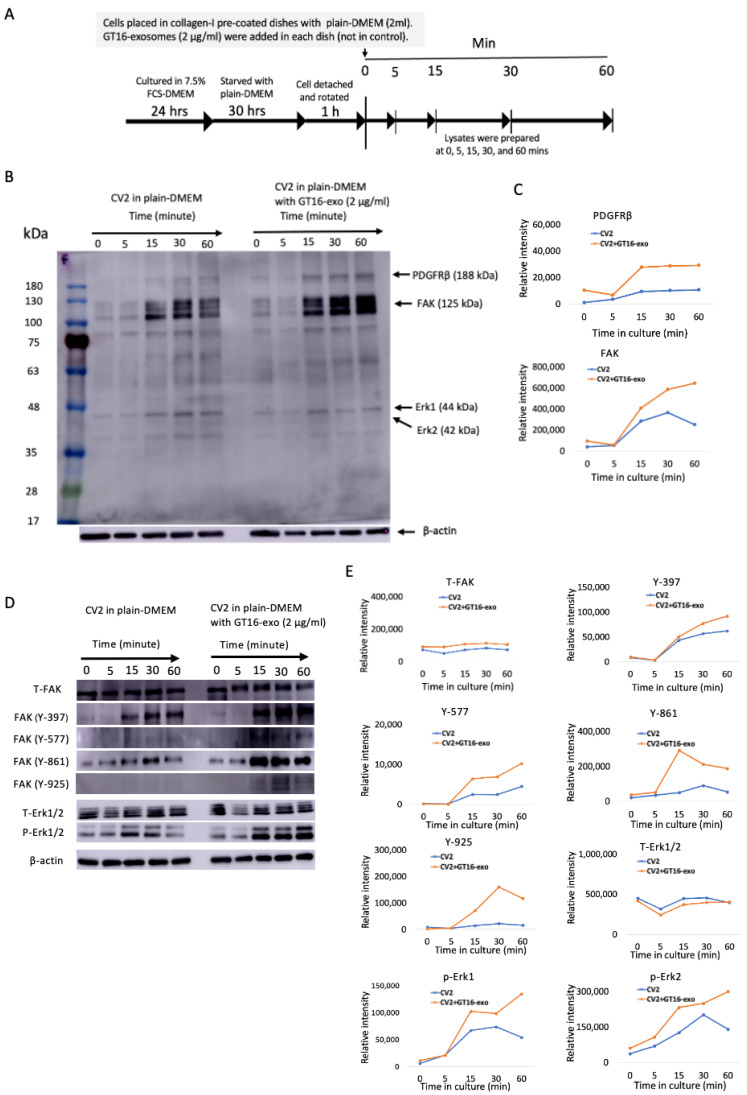
Effects of EVs on cell adhesion signals during CV2 cell adhesion to collagen-I. Tyrosine-phosphorylated signaling protein levels in CV2 cells were increased after treatment with GT16 cell-derived EVs. (**A**) A schema for preparing CV2 cell lysates during their adhesion to collagen-I at different time points in the presence or absence of EVs released from GT16 cells. CV2 cells were placed in collagen-I-precoated dishes (6-cm) with FCS-free DMEM (2 mL) and incubated for 0~60 min at 37 °C in the presence or absence of EVs (4 μg) derived from GT16 cells. After incubation, the cells were lysed at the indicated time points using the lysis buffer. (**B**) The prepared lysates, at several time points, were applied to SDS-PAGE (4 μg/well) to separate proteins. Subsequently, IB was performed to observe the tyrosine phosphorylation levels of different signaling proteins with an anti-phosphotyrosine Ab, PY20. (**C**) The intensities of the obtained bands in (**B**) are measured and plotted. (**D**) The phosphorylation levels at several sites of FAK and in Erk1 and 2 were examined utilizing the obtained lysates (4 μg/well) from (**A**) by IB. The band intensities in (**D**) were measured using Amersham Imager 680 software version 2.0 and are plotted in (**E**). Representative results of repeated (at least 3 times) experiments are presented.

To analyze the effect of GD3/GD2(+) EVs on the intracellular signaling of GD3/GD2(-) cells during cell adhesion, the IB of GD3/GD2(-) cells was performed with or without treatment of EVs using Ab PY20. Furthermore, various site-specific Abs were used to detect the phosphorylation levels at specific sites of signaling molecules. After starvation and detaching, GD3/GD2(-) cells were placed in collagen-I-precoated dishes with serum-free DMEM, and then GD3/GD2(+) cell-derived EVs were added. Cell lysates were prepared as shown in Figure 9A and then subjected to IB. Phospho-tyrosine bands were almost similar until 5 min in both EV+ and EV- conditions (Figure 9B). But after 15 min, bands were stronger in the right panel where cells were treated with GD3/GD2(+) EVs than the left that was not treated. The intensity of two major bands, platelet-derived growth factor receptor β (PDGFRβ) and focal adhesion kinase (FAK) in Figure 9B, are measured and plotted in Figure 9C. Lysates were also utilized to detect the phosphorylation levels at different sites of FAK and in Erk1 and 2. Higher phosphorylation was observed at Y-397, 577, 861, and 925 of FAK and also in Erk1 and 2 in the EV-treated panel (Figure 9D, right) compared to the non-treated one (Figure 9D, left), as shown in Figure 9D,E.

#### 2.6.2. Effects of Anti-Ganglioside Abs on the Action of GD3/GD2(+) Cell-Derived EVs to the Adhesion of the Recipient GD3/GD2(-) Cells

**Figure 10:**
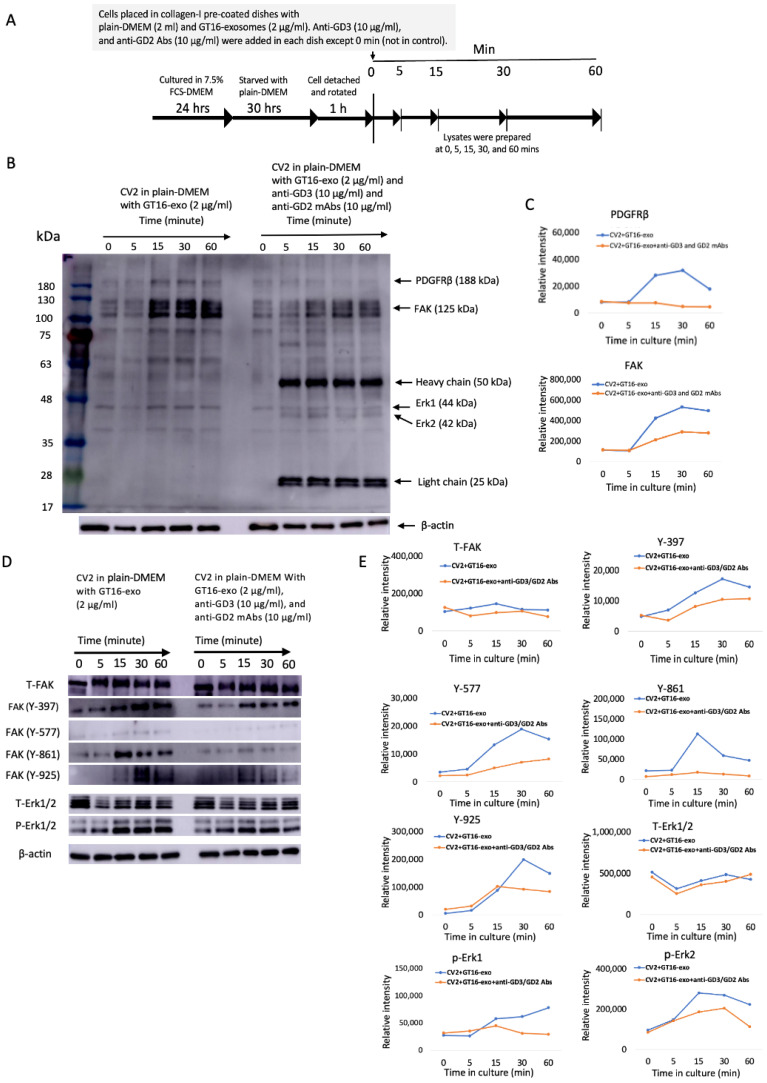
The combined effects of anti-GD3 mAb and anti-GD2 mAb on the action of GD3/GD2(+) cell-derived EVs to the activation of adhesion signals. Tyrosine-phosphorylated protein levels in CV2 cells that were treated with GD3/GD2(+) cell-derived EVs were decreased after the addition of Abs, anti-GD3 mAb and anti-GD2 mAb. (**A**) A schema of preparing CV2 cells treated with GT16 cell-derived EVs to obtain lysates during their adhesion to collagen-I at different time courses in the presence or absence of anti-GD3 and anti-GD2 Abs. GD3/GD2(-) and CV2 cells were placed in collagen-I-precoated dishes (6-cm) in serum-free DMEM (2 mL) with GD3/GD2(+) cell-derived EVs and incubated for 0~60 min at 37 °C in the presence or absence of anti-GD3 and anti-GD2 mAbs. After incubation, cells were lysed at the indicated time points using lysis buffer. (**B**) The prepared lysates were subjected to SDS-PAGE (4 μg/well). Subsequently, IB was performed to examine the tyrosine phosphorylation levels of proteins with Ab PY20. (**C**) The intensity of the obtained bands in (**B**) were measured using Amersham Imager 680 software version 2.0 and plotted. (**D**) The prepared lysates (4 μg/well) as shown in (**A**) were utilized to detect the phosphorylation levels at several sites of FAK and in Erk1 and 2 by IB. The band intensities in (**D**) were measured and are plotted in (**E**). Representative data of repeated experimental results (at least 3 times) are presented.

After starvation of the cultured CV2 cells in serum-free DMEM, cells were detached and transferred into dishes with plain DMEM, and then EVs, anti-GD3 mAb, and anti-GD2 mAb were added. Cell lysates were prepared along the time points indicated in Figure 10A and then used for IB with an anti-phosphotyrosine mAb, PY20. Reduced tyrosine-phosphorylated proteins like PDGFRβ and FAK were detected in CV2 cells treated with GD3/GD2(+) cell-derived EVs and anti-gangliosides mAbs, compared with those treated with EVs alone, as shown in Figure 10B. Phosphorylation at Y-397, 577, 861, and 925 of FAK and in Erk1 and 2 in CV2 cells was decreased after combined treatment with anti-GD3 and anti-GD2 mAbs (Figure 10D). The phosphorylation of Erk1 and 2 were also analyzed using the prepared lysates, and lowered phosphorylation was found after 15 min of the addition of the anti-ganglioside antibodies (Figure 10D), indicating the roles of gangliosides on EVs in their actions.

## 3. Materials and Methods

### 3.1. Cell Lines and Culture

The cell lines used in this study were GT16 and CV2/CV3 as a representative line of GD3/GD2-positive lines (GT16, GT18, GT29) and GD3/GD2-negative lines (CV2, CV3, CV5), respectively [40]. The GD3/GD2-expressing cell lines were generated through the transfection of GD3 synthase cDNA [41] and the neo-resistant gene into the human glioma cell line U-251MG, obtained from the JCRB Cell Bank in Osaka, Japan. The GD3/GD2-non-expressing control lines were also generated by the transfection of the neo-resistant gene alone. The cells were cultured in Dulbecco’s modified Eagle’s medium (DMEM) supplemented with 7.5% fetal calf serum (FCS) and G418 (400 μg/mL) at 37 °C in a humidified incubator containing 5% CO_2_.

### 3.2. Antibodies and Reagents

#### 3.2.1. Antibodies

The anti-GD2 monoclonal antibody (mAb), 220-51, was generated in our group [42], and the anti-GD3 mAb, R24 was generously supplied by Lloyd J. Old at the Memorial Sloan Kettering Cancer Center, New York, USA. All other antibodies were procured from various commercial sources as described below. Fluorescein isothiocyanate (FITC)-conjugated goat anti-mouse IgG (H + L) (Cat. no. 55514) was purchased from Cappel (Durham, NC, USA), and the horseradish peroxidase (HRP)-conjugated anti-mouse IgG antibody (Cat. no. 7076S), HRP-conjugated anti-rabbit IgG antibody (Cat. no. 7074S), anti-Erk1/2 (p44/42 MAPK) (Cat. no. #9102), and anti-p-Erk1/2 (P-p44/42 MAPK) (Cat. no. #9101) were purchased from Cell Signaling Technology (Danvers, MA, USA). Mouse anti-integrin β1 (Cat. no. sc-13590), anti-integrin α2 (Cat. no. sc-74466), anti-integrin α3 (Cat. no. sc-13545), anti-integrin α4 (Cat. no. sc-365569), anti-integrin α5 (Cat. no. sc-376199), anti-integrin α6 (Cat. no. sc-13542), rabbit anti-integrin αv (Cat. no. sc-9969), mouse anti-phosphotyrosine antibody, PY20 (Cat. no. sc-508), and site-specific antibodies of FAK like anti-phospho-FAK Y-397 (Cat. no. sc-81493) and Y-925 (Cat. no. sc-11766) were purchased from Santa Cruz Biotechnology (Santa Cruz, CA, USA). Anti-p-FAK (Y-576, 577, 861) was from Cusabio Technology LLC, TX, USA, and anti-FAK (total) (Cat. no. bs-1340R) was obtained from Bioss Antidodies (Woburn, MA, USA). Anti-PDGFRβ (D-6) (Cat. no. sc-374573) and anti-p-PDGFRβ (Y-716) (Cat. no. sc-365464) were also purchased from Santa Cruz Biotechnology. Alexa fluor 568-conjugated goat anti-mouse IgG antibody (Cat. no. ab175473) and Alexa fluor 488-conjugated goat anti-rabbit IgG antibody (Cat. no. ab150077) were purchased from Abcam Limited (Cambridge, UK).

#### 3.2.2. Reagents

The PS Capture^TM^ Exosome Flow Cytometry Kit (Cat. no. CC050), Tim4-beads (Cat. no. 297-79701), mouse Tim4/human Fc chimera (recombinant) (Cat. no. 081-10261), 4% paraformaldehyde phosphate-buffered solution (Cat no. 163-20145), 2-amino-2 hydroxymethyl- 1, 3-propanediol (Cat. no. 201-062 3), G418 (Cat. no. 076-05962), N, N, N^1^, N^1^-tetramethylethylenediamine (Cat. no. 205-06313), ImmunoStar^TM^ LD (Cat. no. 290–69904) detection kit, sodium hydrogen carbonate (Cat. no. 191–01305), sodium dodecyl sulfate (Cat. no. 196–08,675), and ammonium peroxodisulfate (Cat. no. 016-20501) were purchased from Fujifilm Wako (Osaka, Japan). Dulbecco’s modified Eagle’s medium (DMEM) (Lot. no. RNBK9922), collagen-I (Cat. no. CC050), Tween20 (Cat. no. P2287), and the PKH26 red fluorescent cell linker mini kit (MINI26) were purchased from Sigma-Aldrich (St. Louis, MO, USA). Protease Inhibitor Mixture (Cat. no. 539131) was procured from Calbio-chem (San Diego, CA, USA), and the cell lysis buffer (Cat. no. 9803S) was from Cell Signaling. SsoAdvanced^TM^ Universal SYBR Green Supermix (x2) (Cat. no. 172-5271) was purchased from Bio-Rad (Hercules, CA, USA). The reverse transcriptase enzyme, M-MLV (Cat. No. 28025-013), and 0.1M DTT (Cat. no. 11896744) were purchased from Invitrogen (Burlington, ON, Canada), and dNTP-Mix (Cat. no. 20912) was from QIAGEN (Hilden, Germany). Additionally, 5× Green GoTaq reaction buffer (REF M791B) was purchased from Promega (Madison, WI, USA), and Gene ladder 100 (Cat. no. 316-06915) was from Nippon Gene, Tokyo, Japan. FastGene BlueStar prestained protein marker (Cat. no. MWP03-8) was obtained from Nippon Genetics Europe GmbH (Duren, Germany) and phenylmethylsulfonylfluoride (PMSF) (Cat. no. 10837091001) was provided by Roche Diagnostics GmbH (Mannheim, Germany). ProLong^TM^ glass antifade mount reagent (P36982) was purchased from Thermo Fisher Scientific, Life Technologies Corporation (Waltham, MA, USA). The Pierce^TM^ BCA Protein Assay kit (REF 23228) was from Thermo scientific. Protogel/30% (*w*/*v*) Acrylamide (EC-890) was purchased from National diagnostics (Atlanta, GA, USA), and Corning ITS^TM^ premix universal culture supplement (354352) was procured from Thermo fisher Scientific. All other standard reagents were as described previously [39,40].

### 3.3. Exosome Isolation from Cell Culture Supernatants and Quantification

About 80–90% confluent cells in 15 cm dishes were washed three times with cold 1× PBS. Subsequently, they were cultured for 48 h in DMEM supplemented with 1% ITS premix. Then, the cultured supernatants were transferred to 50 mL Falcon tubes and then centrifuged for 10 min at 4 °C and 500× *g*. Following subsequent centrifugation for 25 min at 4 °C and 20,000× *g*, supernatants were collected and underwent filtration using 0.22 μm Sartolab filters (RF-150) (Zartorius, Helsinki, Finland). The clarified samples were then transferred into Beckman polypropylene ultracentrifuge tubes (Beckman Coulter, Brea, CA, USA). Then, the samples were centrifuged at 175,000× *g* and 4 °C for 84 min employing the Beckman SW32Ti rotor (Kent, MI, USA). Following the removal of supernatants and vortexing, the resulting sediments containing exosomes were resuspended in cold PBS. The samples were then subjected to a second round of centrifugation at 175,000× *g* and 4 °C for another 84 min. Following the removal of supernatants, the samples were slightly vortexed, and 200 μL of cold PBS was added. Afterward, the samples were either used immediately or preserved at −80 °C after being aliquoted. The isolated exosome sample in PBS was utilized for quantifying the protein concentration using the Pierce BCA Protein Assay Kit (Thermofisher Scientific).

### 3.4. Data-Dependent LC-MS/MS Analysis

EVs isolated by ultracentrifugation methods as mentioned above were lyophilized, digested, and desalted according to PTS methods [43]. LC-MS/MS analysis of the resultant peptides was performed on a nano-flow reverse phase liquid chromatography connected to a Q-Exactive Orbitrap mass spectrometer (Thermo Fisher Scientific) through a nano-electrospray ion source (AMR Inc., Tokyo, Japan) [44]. The peptides were separated on a 125 mm C18 reversed-phase column with an inner diameter of 100 µm (Nikkyo Technos, Tokyo, Japan) with a linear 5–40% acetonitrile gradient for 0–100 min, followed by an increase to 95% acetonitrile for 5 min. The mass spectrometer was operated in data-dependent acquisition mode. MS1 spectra were measured with a resolution of 70,000, an automatic gain control (AGC) target of 3 × 10^6^, and a mass range of 350–1800 *m*/*z*. HCD MS/MS spectra were acquired with an AGC target of 1 × 10^5^, an isolation window of 2.0 *m*/*z*, a maximum injection time of 60 ms, and a normalized collision energy of 27. Dynamic exclusion was set to 10 s. Raw data were directly analyzed against the Swiss-Prot database restricted to Homo sapiens and cRAP for contaminant databases using Proteome Discoverer version 2.4 (Thermo Fisher Scientific) with the SEQUEST search engine. The search parameters were as follows: (a) trypsin as an enzyme with up to two missed cleavages; (b) precursor mass tolerance of 10 ppm; (c) fragment mass tolerance of 0.02 Da; (d) carbamidomethylation of cysteine as a fixed modification; and (e) acetylation of protein N-terminus and oxidation of methionine as variable modifications. Peptides were filtered at a false discovery rate (FDR) of 1% using the Percolator node. Label-free quantification was performed based on the intensities of precursor ions using the precursor ion quantifier node. Normalization was performed such that the total sum of abundance values for each sample over all peptides was the same.

Data availability: LC-MS data that support the findings of this study have been deposited to jPOST with the identifier JPST003411 (PXD056795). (20240731_matrix6_Final.xlsx).

### 3.5. Flow Cytometry

The expression levels of the gangliosides (GD3 and GD2) and integrins (β_1_, α_2_, α_3_, α_4_, α_5_, α_6_, and α_v)_ on the cell surface were analyzed by flow cytometry using an Accuri^TM^ C6 flow cytometer (BD Biosciences, Franklin Lakes, NJ, USA), as previously described [45]. Briefly, following the trypsinization of cultured cells, a cell suspension containing 3 × 10^5^ cells was incubated with primary antibodies in PBS for 60 min on ice. After washing twice with PBS, the cells were stained with diluted (1:100) secondary antibodies, FITC-conjugated goat anti-mouse IgG (H + L) (Cappel), in PBS for 45 min on ice in a dark place. Afterward, the expression levels were analyzed by a flow cytometer. All control samples were prepared using non-relevant Abs with the same subclasses as individual primary antibodies. After flow cytometry, the obtained data were analyzed using the CFlow plus^TM^ program. Regarding the isolated exosomes, the expression levels of GD3, GD2, and integrins on exosomes were examined using Tim4-beads. Exosomes (0.25 μg) were incubated with PS-capture^TM^ Tim4-beads (Fujifilm Wako) at room temperature for 1 h with gentle vortexing every 10 min. After the formation of the Tim4-beads–exosome complex, unbound exosomes were washed out twice using a magnetic stand (290-35591, Fujifilm Wako), and then primary antibodies were added in proper dilution (1:100) against GD3, GD2, or integrins and incubated on ice for 1 h with shaking every 10 min. The unbound antibodies were washed out twice. Then, the secondary antibodies, FITC-conjugated goat anti-mouse IgG (H + L) (Cappel), were applied at a proper dilution (1:100) for 30 min with gentle shaking every 10 min on ice in dark place. After washing twice, Tim4-beads–exosome complexes were subjected to flow cytometry.

### 3.6. Real-Time Reverse Transcription-Quantitative PCR (RT-qPCR)

Total RNA extraction from cells and exosomes was performed with TRIzol^TM^ reagent (Invitrogen), and a cDNA template was synthesized from the total RNA using the MMLV reverse transcriptase kit (Invitrogen) as described previously [45]. In brief, 2 μg of total RNA was used for cDNA synthesis with MMLV reverse transcriptase. Synthesized cDNA (4 ng) was amplified in a 20 μL total reaction volume containing 10 μL of the SsoAdvanced^TM^ Universal SYBR green Supermix^TM^ qPCR kit (Bio-Rad Laboratories, Hercules, CA, USA) and 1 μL each of 5 μM primer. PCR was performed with designed primer sequences for the genes as follows: *ST8SIA1*—forward (5′-GGAAATGGTGGGATTCTGAAG-3′), reverse (5′-TGACAAAGGAGGGAGATTGC-3′) (amplified size 49 bp); *B4GALNT1*—forward (5′-CCAACTCAACAGGCAACTACAA-3′), reverse (5′-ATGTCCCTCGGTGGAGAAC-3′) (amplified size 53 bp); *β-actin*—forward (5′-CCAACCGCGAGAAGATGA-3′), reverse (5′-CCAGAGGCGTACAGGGATAG-3′) (amplified size, 59 bp); *hGAPDH*—forward (5′-ACTTCAACAGCGACACCCAC-3′), reverse (5′-CAACTGTGAGGAGGGGAGAT-3′) (amplified size 213 bp). The PCR program was carried out with initial denaturation at 95 °C for 30 s, followed by 40, 34, or 32 cycles of amplification (95 °C for 5 s, 58 °C for 30 s, and 65 °C for 5 s).

### 3.7. Immuno-Cytostaining

GT16 and CV2 cells (2 × 10^4^) were seeded in collagen-I-pre-coated glass-bottomed dishes (Iwaki, Tokyo, Japan) and incubated in DMEM supplemented with 7.5% serum at 37 °C for 24 h. After washing with cold PBS, 4% paraformaldehyde in PBS was used for 10 min at room temperature to fix the cells on the glass bottom. Then, the fixed cells were blocked with 5% BSA in PBS for 1 h at RT. Immunostaining was performed using the following primary antibodies: mouse anti-GD3 mAb (R24), mouse anti-GD2 mAb (220-51), and rabbit anti-integrin β_1_ polyclonal Ab at appropriate dilution in 2% BSA/PBS for 1 h at RT. After washing with 1% BSA/PBS, cells were incubated with Alexa fluor 568-conjugated goat anti-mouse IgG (Invitrogen) and Alexa fluor 488-conjugated goat anti-rabbit IgG (Invitrogen) in 2% BSA/PBS for 1 h at RT. After being washed properly, cells were mounted with Pro-Long anti-fade reagent (Thermo Fisher Scientific). Then, the cells were analyzed by a confocal microscope (Fluoview FV10i; Olympus, Tokyo, Japan) or an all-in-one fluorescence microscope, the BZ-810, KEYENCE (Tokyo, Japan).

Regarding exosome staining, exosome suspension (in PBS) was placed in a glass-bottom dish precoated with Tim4-Fc and incubated for 30 min at RT. Then, the dish was washed once with PBS, and 4% paraformaldehyde in PBS was used for 10 min to fix the exosomes on the glass bottom. Then, the sample was stained with antibodies following the protocol mentioned above for cells and finally analyzed under confocal microscope (Fluoview FV10i).

### 3.8. Adhesion Activity of Cells to CL-I

Cell adhesion activity to collagen-I (CL-I) was analyzed by a real-time cell electronic sensing system^TM^ (RT-CES) (Wako), as described previously [26]. An E-plate or microplate with an electronic sensor in the bottom was used to measure the cell adhesion activity. Microelectronic cell sensor arrays integrated at the bottom of the microplates (E-Plate (16×) from ACEA Biosciences Inc., San Diego, CA, USA) conveyed information about increased electrical resistance (cell index), reflecting the increase in cell adhesion. For the initialization of the assay, E-plates were coated with CL-I at a concentration of 5 μg/mL in PBS (100 μL/well) at RT for 1 h. Subsequently, the plates were blocked with 1% BSA/7.5% FCS in DMEM (100 μL/well) at RT for additional 1 h. Following blocking and washing, cells (1 × 10^4^) were seeded into each micro-well of the microplates containing the culture medium, 7.5% FCS-DMEM. To explore the effect of anti-GD3/GD2 mAbs or anti-integrin β_1_/α3 Abs on the adhesion activity of the cells, they were introduced with cells along with exosomes at time 0. The changes in cell adhesion activity were continuously monitored and are expressed in terms of the cell index (CI).

### 3.9. Lysate Preparation from Cells and Exosomes

The procedure for cell lysate preparation was as described previously [36,43]. In brief, after washing the cultured cells (70–80% confluent) three times in 6 cm dishes with cold PBS, lysis buffer (comprising 20 mM Tris-HCl, 1 mM EGTA, 1 mM Na_2_-EDTA, 150 mM NaCl, 1% Triton X-100, 1 mM β-glycero-phosphate, 2.5 mM sodium pyrophosphate, 1 mM Na_3_VO_4_, and 1 μg/mL leupeptin) (Cell Signaling) was added for cell lysis, and the dish was kept on ice for 5 min. After 5 min, the cells were scraped, and lysates were collected. Next, 1 mM PMSF and Protease Inhibitor Mixture^TM^ (Calbiochem) were added to the dish before collection. The obtained lysates were then subjected to centrifugation at 3000 rpm (Kubota 3740^TM^, Tokyo, Japan) for 4 min at 4 °C to remove insoluble cell debris. After repeated centrifugation, the resulting supernatants and lysates were used to determine the protein concentration using the DC protein assay kit (Bio-Rad). Concerning exosomes, the isolated exosomes in PBS were utilized for the preparation of exosome lysates. Lysis buffer (2×) was used to lyse exosomes by adding it at a 1:1 ratio on ice for 5 min, and it was thoroughly mixed by pipetting. The protein concentration of the prepared exosome lysates was determined using a BCA protein assay kit (Thermo Scientific).

### 3.10. Immunoprecipitation (IP)

Cultured cells (70–80% confluent) in 6 cm dishes were washed three times with PBS and lysed on ice to prepare cell lysates as mentioned above. After removing the insoluble cell debris through repeated centrifugation, the supernatants (cell lysates) were used for immunoprecipitation with mouse anti-integrin β_1_ mAb (Santa Cruz Biotechnology) at 4 °C overnight with rotation. Protein G Sepharose 4 fast flow^TM^ beads (GE Healthcare, Uppsala, Sweden) were used to capture the immune complex. After washing the beads with the immune complex, a sodium dodecyl sulfate (SDS) sample buffer (2×) with 2ME was added and boiled at 95 °C for 5 min. Then, the immunoprecipitated complex was collected and subjected to SDS–polyacrylamide gel electrophoresis (SDS–PAGE).

### 3.11. Immunoblotting (IB)

The proteins in prepared lysates or IP complexes were separated through SDS–PAGE with 10% acrylamide gels as described previously [36,46]. A sample buffer (2×) comprising 125 mM Tris-HCl (pH 6.8), 4% SDS, 20% glycerol, 4% 2-mercaptoethanol, and 0.1% bromophenol blue× was mixed with lysates at a 1:1 ratio and then boiled at 95 °C for 5 min prior to separation by SDS-PAGE. The separated proteins in gels were subsequently transferred onto an Immobilon-P membrane (EMD Millipore, Burlington, MA, USA), and blots were blocked for 1 h or overnight with 5% skim milk in PBST (0.05% Tween-20 in PBS) or with 5% BSA in PBST. The primary antibody reaction with proper dilution was carried out for 1 h at RT or overnight at 4 °C. After washing with PBST, the reaction with the HRP-conjugated secondary antibody was performed for 1 h at RT in the dark. After washing with PBST, protein bands were visualized using ImmunoStar^TM^ LD detection kits (Wako). The resulting band images were analyzed with Amersham Imager (Model 680, software version 2.0, GE Healthcare, Uppsala, Sweden).

### 3.12. Statistical Analysis

Statistical analyses were conducted as previously described [39,40]. The obtained data are presented as the mean ± SD. An unpaired two-tailed Student’s *t*-test and a two-way ANOVA with the Tukey post hoc test were used to compare mean values. The specific details regarding these results are outlined in the legend accompanying each figure. *p* values of <0.05 were deemed as statistically significant. The analysis was performed using R software (version 3.6.3).

## 4. Discussion

The main role of exosomes has been claimed to be mediators for cell-to-cell communication [28,47]. Through our experiences in the function analyses of cancer-associated ganglioside-expressing cell-derived exosomes in melanomas, exosomes seem to exert almost all functions of the gangliosides, which have been reported to be effects of cancer-associated gangliosides expressed on the cell membrane [26,39]. This is also the case in gliomas, as reported by our group [38,40]. Function analyses of exosomes revealed their effects on the enhancement of malignant phenotypes when added to cancer-associated ganglioside-negative target cells. While there have been many studies on exosomes derived from gliomas [48], no reports on gangliosides in glioma exosomes could be found. Thus, we analyzed roles of GD3/GD2 in exosomes for the first time, exhibiting enhanced cell growth, invasion, mobility, and cell adhesion by the addition of exosomes from GD3/GD2-expressing glioma cells [40]. Here, we analyzed mechanisms by which GD3/GD2-positive exosomes exert their roles by elucidating molecular profiles contained in EVs from GD3/GD2-positive and -negative cells, as shown in Figure 1, clarifying the spatio-temporal dynamics of EV actions, and exhibiting the collaboration of integrins and gangliosides. Intriguingly, not only cancer phenotypes but intracellular signaling in the targets was also regulated by exosomes via the collaboration of integrins and gangliosides on EVs, suggesting their roles in inter-cellular conflict and competition among heterogenous cancer cell populations [39]. In addition, detailed compositions of disialyl gangliosides, such as isoforms of ceramides and 9-*O*-acetyl derivatives, were not examined this time. These fine ganglioside compositions and related miRNAs remain to be elucidated. Furthermore, the possibility of the presence of galectins in the vicinity of gangliosides/integrin complexes on the exosomes and its influence should be considered in the future [49].

Since the mechanisms for the actions of exosomes to target cells have been almost unknown, the results reported here might provide valuable insights into understanding how they exert their roles around and inside target cells, i.e., the attachment of exosomes on the surface of cells, the roles of gangliosides on exosomes in attachment, the time course of transferred/expressed gangliosides, the fates of exosomes inside of target cells, and the lasting effects of one-shot added exosomes. In particular, adhesion signals mediated by integrins were definitely enhanced by the cluster formation of integrins with gangliosides on exosomes, and they were clearly suppressed by anti-ganglioside antibodies both in RT-CES experiments (Figure 8) and in cell signaling analyses with IB (Figure 10). These results suggest that gangliosides such as GD3 and GD2 exert important roles in the targeting of exosomes to recipient cells via some receptors on the cell surface such as the extracellular matrix [50]. The suppressive effects of anti-integrin β1 Ab on exosome actions supported this concept, while anti-integrin α3 Ab showed minimal effects, suggesting differential binding of these two anti-integrin Abs on the heterodimer complex. The fact that many signals were activated after 5~15 min of exosome treatment suggests that these signals might be transduced via some receptors on the surface of target cells [51]. Although the cellular receptors on the target cell surface remain to be investigated, the molecular clustering of integrins and gangliosides on exosome membranes [39] as well as cell membrane microdomains [52] should become a therapeutic target in anti-cancer strategies.

From a limited range of evidence, FAK seems to be the most reactive signaling molecules during cell growth and/or adhesion stimuli induced by added exosomes, as elucidated by the phospho-tyrosine analyses and shown in Figure 9 and Figure 10. Simultaneously, PDGFR and ERK1/2 also showed increased phosphorylation within 5~15 min of stimulation. Furthermore, this increased phosphorylation was suppressed by anti-ganglioside mAbs. This result suggests that integrins on exosomes are playing essential roles together with gangliosides, at least in the initial phase of exosome actions. Although there were no clear differences in the expression levels of integrins on/in the exosomes from GD3/GD2-positive and GD3/GD2-negative cells as well as on the cell surface (Figure 1 and Figure 4), the presence of GD3/GD2 should enhance the functions of integrins by physical association with each other (Figure 7), leading to the strong phosphorylation of FAK at many tyrosine residues on it in recipient cells (Figure 9D and Figure 10D).

Although there have been many reports on the involvement of the integrin–FAK axis in exosome functions [53,54,55], it is not clear how integrins on the exosome surface can trigger activation signals in target cells. How exosomes exert these signaling pathways in the target cells via integrin–ganglioside clusters on them remains to be elucidated. The implication of site-specific tyrosine-phosphorylation of FAK in phenotypic changes of the target cells is also an intriguing issue. The autophosphorylation site (Y397) appears to be the main binding site for Src, etc., and Y861 phosphorylation was reported to be necessary for cell migration. The phosphorylation of Y925 is reported to be associated with integrin adhesion [56]. All these phosphorylation reactions were enhanced 15 min after addition of EVs (Figure 9). Furthermore, the addition of anti-ganglioside mAbs resulted in the suppression of phosphorylation bands, as shown in Figure 10, suggesting the crucial roles of gangliosides on EVs in the functioning of EVs.

Since there have been no comprehensive reports on the fate and intracellular location of exosomes after being incorporated into target cells, the results reported in this study, i.e., the time course of ganglioside synthase gene expression and ganglioside expression and intracellular distribution, might provide an impact on the analysis of exosome behaviors. At this moment, many investigations remain to be performed with appropriate concentration and the continuous presence of exosomes similar to those in our bodies.

When compared with the actions of exosomes from GD2-positive melanoma cells [39], enhancements to cell adhesion in gliomas were not so marked as in melanomas, suggesting that the mechanisms of exosome functions might not be exactly the same because of different cancer types or different ganglioside composition, as reported previously [26]. There are many reports on the mechanisms for the generation of exosomes, from endosome formation to cargo loading and fusion with cell membrane to be secreted [57,58]. Generally, the main executing molecules for such processes have been studied [59,60], but actual exerting molecules for exosome generation under cancer-associated gangliosides have never been reported. To understand the specific functions of exosomes derived from GD3 and/or GD2-expressing cells including melanomas and gliomas, we need to consider the ganglioside-oriented loading of cargoes into exosomes. This issue should be one of the most crucial subjects in this study. The roles of cell membrane microdomains for the generation of frames of exosomes quickly come up in as an idea in [61], but it needs sophisticated skill and remains an urgent issue to be solved.

Recently, the clinical application of cancer-derived exosomes has attracted the attention of many researchers. In particular, the roles of exosomes in the construction and development of cancer stem cells seem quite fascinating and suggest their possible target of cancer therapeutics [62,63]. Reports that ganglioside GD3 and/or GD2 confer cancer-stem-like features in gliomas [64] or breast cancers [22], respectively, make us excited, and we urge the promotion of significant advances.

## Data Availability

Data are contained in the article and are available upon request from the corresponding authors. LC-MS data that support the findings of this study have been deposited to jPOST with the identifier JPST003411 (PXD056795). (20240731_matrix6_Final.xlsx).

## Abbreviations

EVs	extracellular vesicles
PDGF	platelet-derived growth factor
FAK	focal adhesion kinase
Erk1/2	extracellular signal-regulated kinase 1/2
ST8SIA1	alpha-N-acetylneuraminide alpha-2,8-sialyltransferase
B4GALNT1	N-acetyl-galactosaminyltransferase 1
DMEM	Dulbecco’s modified Eagle’s medium
FCS	fetal calf serum
FITC	fluorescein isothiocyanate
HRP	horseradish peroxidase
GD3	Neu5Acα2,8Neu5Acα2,3Galβ1,4Glc-ceramide
GD2	GalNAcβ1,4(Neu5Acα2,8Neu5Acα2,3) Galβ1,4Glc-ceramide)
BSA	bovine serum albumin
SDS-PAGE	sodium dodecyl sulfate–polyacrylamide gel electrophoresis
RT-CES	real-time cell electronic sensing system
